# Multi-dimensional Perspective Pharmaceutical Evaluation: A Path to Enhancing Healthcare Decision-Making in Real-World

**DOI:** 10.34172/ijhpm.2024.8295

**Published:** 2024-03-05

**Authors:** Jinmiao Lu, Xiaohua Ying, Zhiping Li

**Affiliations:** ^1^Department of Clinical Pharmacy, National Children’s Medical Center, Children’s Hospital of Fudan University, Shanghai, China.; ^2^NHC Key Laboratory of Health Technology Assessment, Department of Health Economics, School of Public Health, Fudan University, Shanghai, China.

## Dear Editor,

 China is one of the world’s largest producers of active pharmaceutical ingredients and generic drugs.^1^ In 2021, the Chinese government established a comprehensive evaluation system that defines the value of pharmaceuticals across six dimensions, including safety, efficacy, cost-effectiveness, appropriateness, accessibility, and innovation.^2^ Policy-makers and clinical specialists consider six criteria and their relevant sub-criteria in assessing the overall performance of drugs. Firstly, safety aspects encompass adverse drug reactions causing systemic damage and the incidence rate of individual adverse reactions. Secondly, therapeutic effectiveness covers all endpoints employed in high-quality clinical trials. In terms of costs, we take into account fixed, variable, and marginal costs. The evaluation of innovation includes clinical, service, and industrial innovation, assessed through expert conference ratings. Adaptability involves drug technical characteristics, suitability for different populations, and compliance with drug specifications. Lastly, accessibility considerations encompass drug pricing, availability for each patient, and the economic affordability of medications. This comprehensive assessment framework facilitates a thorough understanding of drug performance, supporting more holistic decision-making and medical choices.

 China plans to establish 100 evaluation data sources primarily from designated hospitals, serving as a standard database for real-world rational medication. Continuous collection and aggregation of clinical medication data from designated hospitals are carried out, followed by regular comprehensive assessments. China is conducting drug evaluations in three pilot directions: pediatric medicine, oncology drugs, and cardiovascular drugs, with corresponding technical guidelines being released.^3^ As one of the major players in pediatric drug evaluation, we have developed a rapid assessment method for pediatric medications based on objective models and expert advice.^4^ The objective of drug comprehensive evaluation is to screen the best drugs for China’s healthcare negotiations, healthcare catalogue and National Essential Drug Catalogue. However, one of the challenges in the evaluation process has always been defining pharmaceuticals in the real world.

 The definition of a pharmaceutical product typically Ides its chemical composition, purpose, manufacturer, and batch. Various factors come into play within the category of pharmaceuticals for treating the same disease, encompassing specific production periods, brands from specific manufacturers, and different treatment targets for the same disease. Simultaneously, pharmaceutical prices, quality, supply, and dosage forms from different generic drug manufacturers have been in constant flux.

 In the real world, drug evaluation encounters a diverse array of drugs that do not uniformly fit into the same evaluation or ranking criteria. Here, we propose a classification approach centered on three crucial aspects of drug candidates: the drug’s target receptor, the manufacturer, and various production cycles. This framework enables a more comprehensive assessment of disease-specific drug utility. In the context of our leading role in pediatric medicine evaluation in China, we categorise pharmaceutical assessment into three dimensions (Figure).

**Figure:**
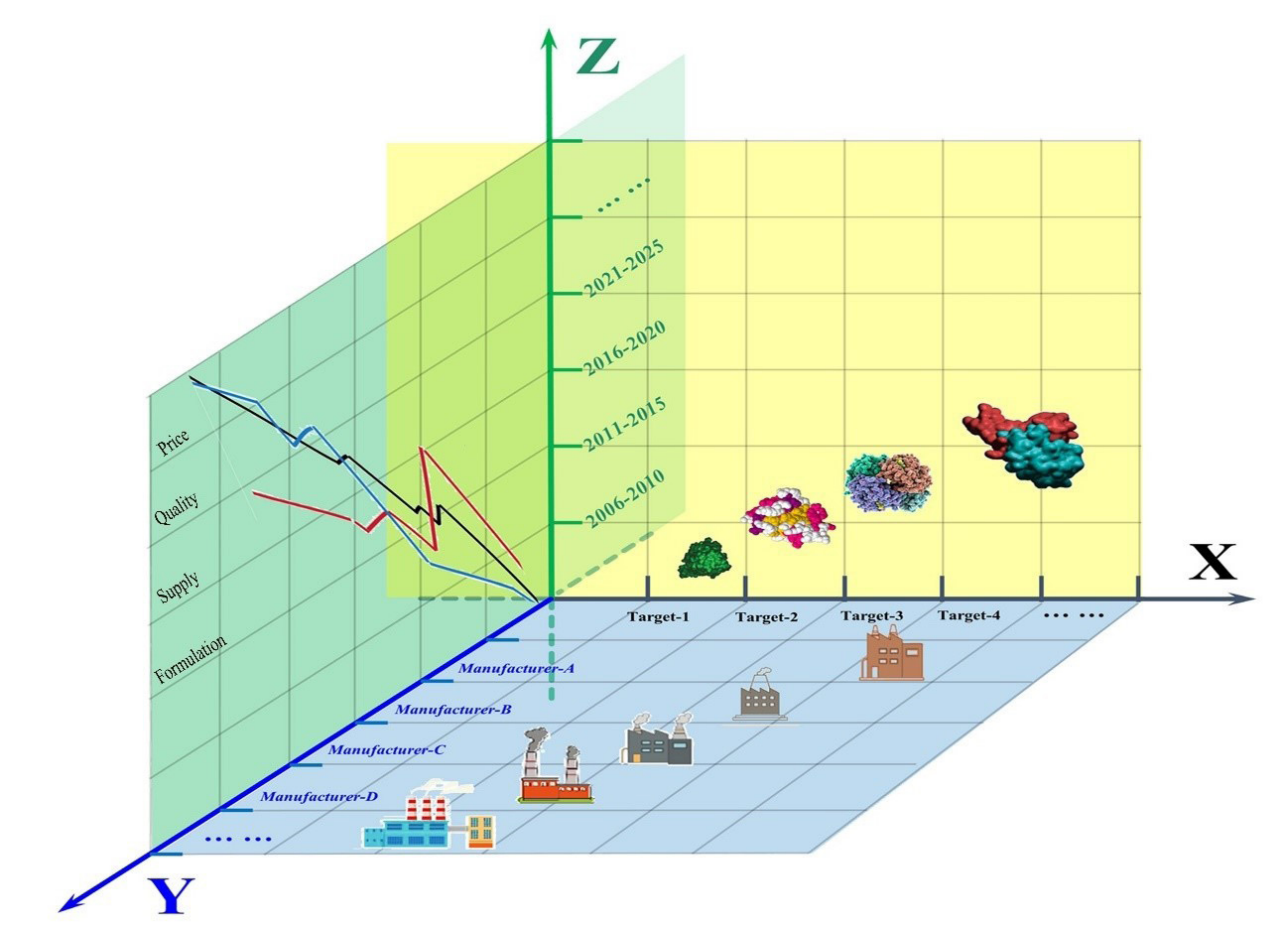
Three-Dimensional Indicators for Drug Classification (X: pharmacologic target receptors; Y: manufacturers; Z: data average over five years, including price, quality level, supply, and formulation changes).

 As shown in the figure, we consider pharmaceuticals targeting the same disease but with varying treatment mechanisms. Second, we examine products from different manufacturers, encompassing ME-TOO drugs (similar to existing ones but not necessarily superior) and ME-BETTER drugs (improved versions of existing treatments). Notably, despite replicating reference drugs, generic and biosimilar drugs may exhibit differences.^5^ Lastly, we acknowledge that the evaluation outcomes of pharmaceuticals at different production stages can diverge due to factors such as pricing and production processes. The persistent challenge in the pharmaceutical industry is ensuring consistent, high-quality drug production over time.

 Compared to countries focusing on a single dimension, typically innovative drugs,^6^ China’s approach introduces some differences regarding the evaluation criteria. In this classification of drugs, you can get a panoramic view of any drug, with the following five advantages:

1. This multi-dimensional perspective improves the understanding of pharmaceuticals for various stakeholders. It gives healthcare professionals and decision-makers a complete view of how different drugs perform in specific medical areas, including their effectiveness, safety, cost-effectiveness, and accessibility. Additionally, it helps regulatory authorities gain better insights into the quality and performance of various brands and manufacturers.
2. This approach fosters competition and innovation in the pharmaceutical industry. Analysing different drugs for the same condition and products from various manufacturers encourages market competition. This competition drives pharmaceutical companies to continually enhance and innovate their drugs to provide more effective, safer, and cost-efficient treatment options. As a result, China’s evaluation model may motivate pharmaceutical firms to innovate and create more competitive drugs, thereby improving drug quality and effectiveness.
3. Multi-dimensional evaluation also leads to more precise treatment choices. Different drugs for the same condition may have distinct mechanisms of action or target different aspects, enabling healthcare professionals to make tailored treatment decisions for individual patients. This personalised approach contributes to better treatment outcomes and reduces unnecessary adverse reactions.
4. The approach benefits pharmaceutical approval and regulation. Multi-dimensional pharmaceutical evaluations offer regulatory authorities comprehensive information, enabling them to comprehend pharmaceuticals’ overall performance better and make informed decisions to ensure patient safety and rational medication use. For instance, considering evaluation results over time can better reflect changes in pharmaceuticals, such as price and manufacturing processes. Limiting the evaluation data to specific timeframes ensures the assessments remain timely and accurate, maintaining their practicality and relevance.
5. The approach supports data-driven decision-making. Regularly assessing pharmaceutical performance at different intervals allows decision-makers to adjust medical policies and medication guidelines based on data, ensuring patients receive the best treatment outcomes. For example, decision-makers can promptly identify differences among generic drugs or disparities among antihypertensive drugs targeting different sites, enabling them to choose the most suitable drugs based on existing data.

In conclusion, China’s pharmaceutical market is vast and highly competitive, necessitating multi-dimensional evaluation to safeguard patient rights and ensure pharmaceutical quality. This approach, considering various dimensions of pharmaceuticals, including treatment targets, manufacturers, and timeframes, contributes to a more comprehensive understanding of pharmaceutical safety and efficacy. It is helpful to the regulation, scientific methodology, and homogenisation of the evaluation process.

## Acknowledgements

 We are indebted to all participants for their consent and co-operation.

## Ethical issues

 Not applicable.

## Competing interests

 Authors declare that they have no competing interests.
